# Change of tourism organizations: Implications from a review of cultural tourism research

**DOI:** 10.3389/fpsyg.2022.1000117

**Published:** 2022-10-05

**Authors:** Ziling Zhang, Muyang Guo

**Affiliations:** ^1^Alliance Manchester Business School, The University of Manchester, Manchester, United Kingdom; ^2^School of Event and Communication, Shanghai University of International Business and Economics, Shanghai, China

**Keywords:** tourism organizational change, change management, implications, brief review, cultural tourism research

## Abstract

Change has been universally acknowledged as the perpetual theme for routine organizational life. As cultural tourism, a major element of global tourism consumption accounting for 40% of tourism employment, is becoming increasingly flourishing and promising, tourism organizations are also obliged to implement a series of organizational changes to adapt to the trending culturalization in the tourism domain. In light of this, this research, by outlining important sub-themes and trends of cultural tourism research, tracks the evolution of cultural tourism as a research field over the previous decades so as to analyze existing interconnections between the systematic review and tourism organizational change. Based on these interconnections, the research also manages to propose several potential implications for tourism organizations to optimize their future implement of daily organizational changes for the sake of adaptative survival and development.

## Introduction

In a business environment that is increasingly volatile and dynamic, organizations strive consistently to adjust and adapt their operations to changing conditions (Hope Hailey and Balogun, 2002). Existing literature on organizational management has widely admitted the centrality of change in organizational life (North, 1996; Orlikowski, 1996; Weick, 1998; Al-Haddad and Kotnour, 2015; Errida and Lotfi, 2021). Thanks to the changing nature of all things in life (James, 1996) as well as the fact that organizations are creations of human agents who are the main source of change seeds (Feldman, 2000), organizational change has been universally accepted as a pervasive norm, an inevitable routine, and a consistent operation (Tsoukas and Chia, 2002). As organizations have been increasingly regarded as an emergent property of change (Errida and Lotfi, 2021), traditional misconception that change exists merely as an exception or an epiphenomenon (Beer and Nohria, 2000) has gradually been weeded out. In other words, change is becoming the only certain stability in organizations, making change management a compulsory course for all kinds of organizations, including tourism organizations, to survive the elusory future (Kickert, 2014; Soulard et al., 2019; Errida and Lotfi, 2020).

In addition to the common rationale for tourism organizations to adapt to changes (i.e., change management is compulsory for all kinds of organizations), the volatility of the tourism context proves to be a more urgent rationale for tourism organizations to change readily (Smith et al., 2014). Specifically, recent decades have witnessed the inextricable link between culture and tourism turning into a specific form of consumption known as cultural tourism (Richards, 2018), which is a significant revolution in the tourism context. Accounting for 40% of the global tourism revenues (UNWTO, 2021), cultural tourism possesses unprecedentedly promising prospects by acquiring its place in the tourism policy of 89% UNWTO member countries around the world (UNWTO, 2018; Petrei et al., 2020; UNESCO, 2022) as well as showing a market volume growth rate up to 130% during the recent 5 years (OECD, 2018; UNWTO, 2021). Thus, it is inevitable for tourism organizations to make some changes to adapt to the rising of cultural tourism.

In light of this, the research first makes a systematic review that summarizes some of the most important sub-themes associated with cultural tourism research. It then analyzes the interconnections between each sub-theme of cultural tourism research and tourism organizational change management, based on which the research finally manages to figure out potential implications for tourism organizations to make and manage changes.

## A review of cultural tourism research and interconnections with tourism organizational change

As cultural tourism is gaining plenty of popularity in both the tourism industry and the tourism literature, it is necessary to have a general understanding on extant cultural tourism research. Therefore, this multi-sectional part first reviews and summarizes cultural tourism literature in terms of such research topics as the current research status, definition, typology, important branches, and optimal development strategies. Then the review presented in each section functions as the primary evidence to figure out the interconnections between cultural tourism research and tourism organizational change (as is shown in Table 2), laying foundations for proposing potential implications in the next part.

### Current status of cultural tourism research

**Figure 1:**
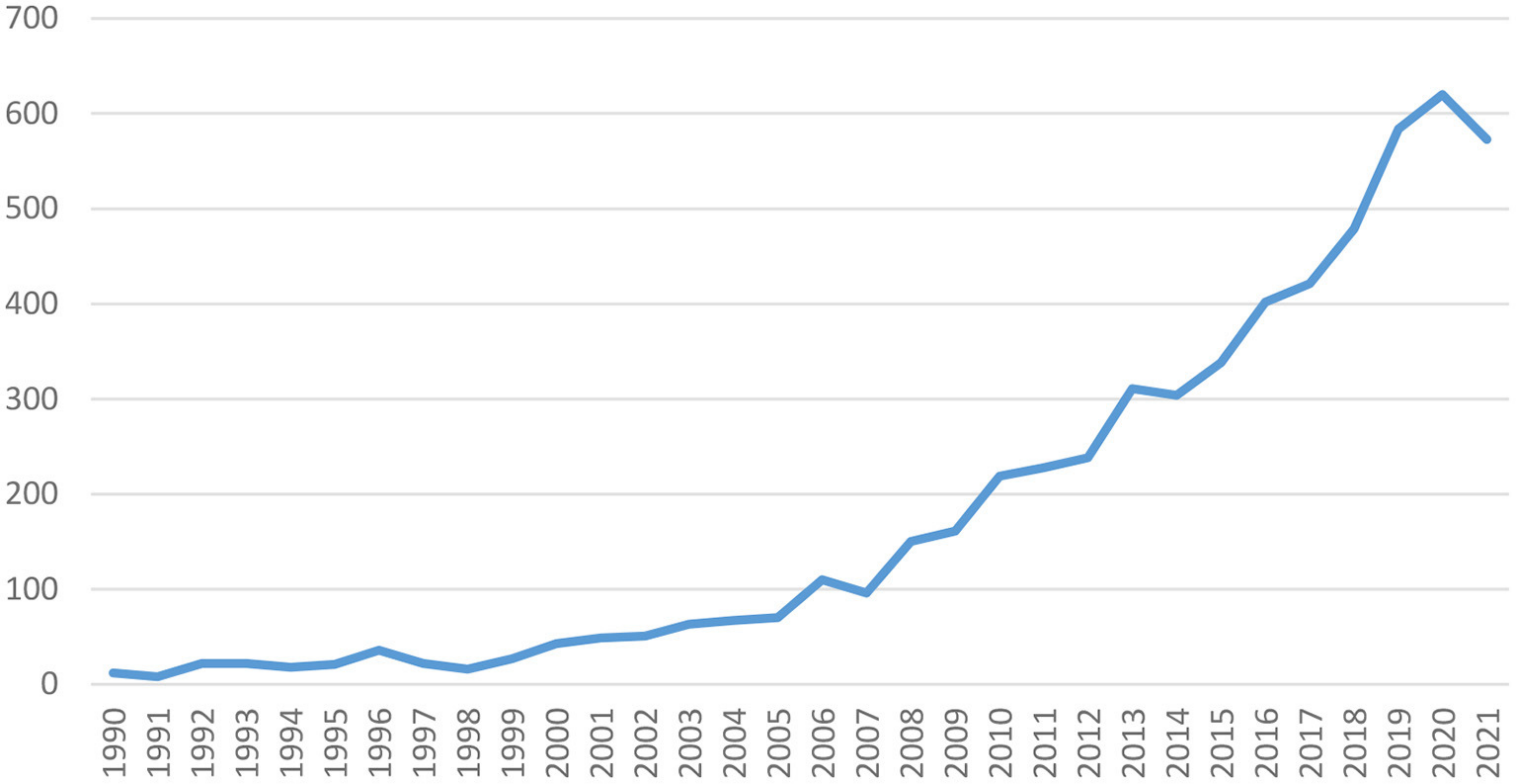
1990–2021 number of cultural tourism publications.

To acquire a better understanding of the current research status of cultural tourism, we adopt a Google Scholar search for the term *cultural tourism* to generate the total number of cultural tourism research publications in each year of 1990–2021. Note that in order to improve the results accuracy, only publications specifically focusing on the term *cultural tourism* (i.e., excluding publications that only mention the full term or part of the term) are included in the samples. As is shown in Figure 1, the recent three decades (1990–2021) have witnessed a significant uptrend on the total volume of cultural tourism research publications. Specifically, growth was particularly sharp between 2005 and 2020.

**Figure 2:**
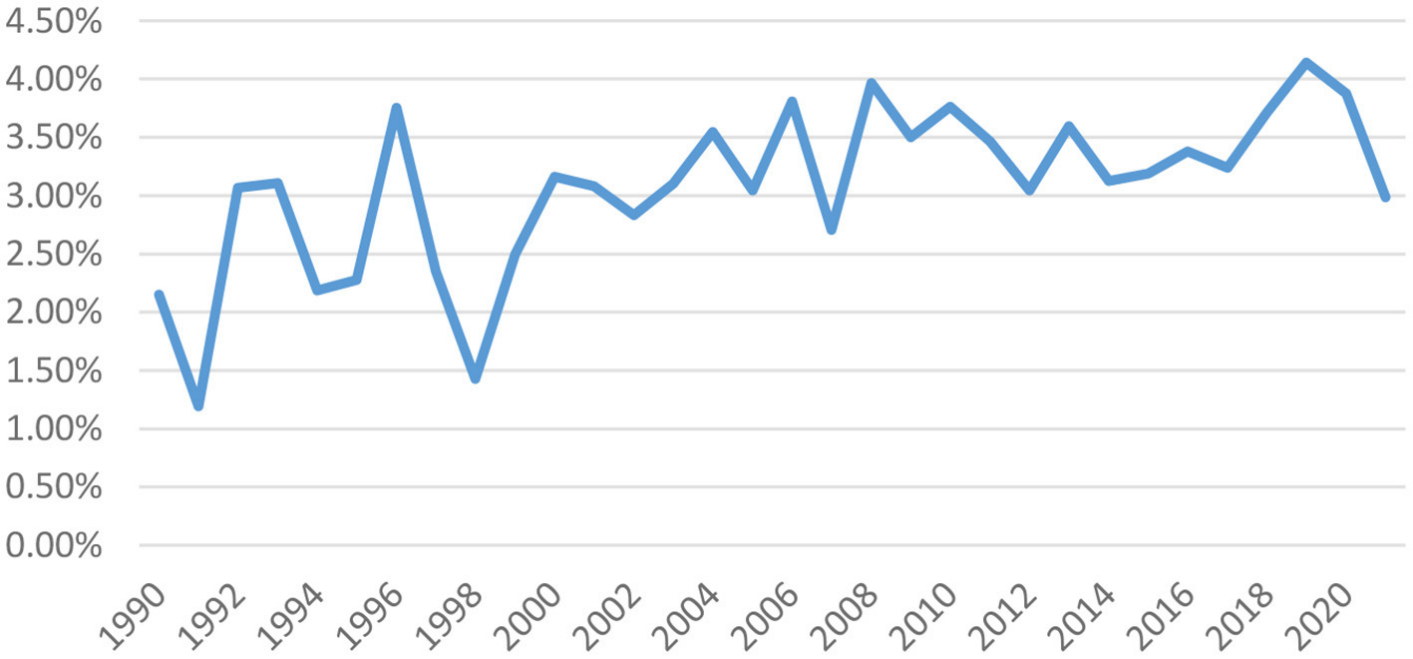
1990–1992 proportion of high-quality publications.

**Figure 3:**
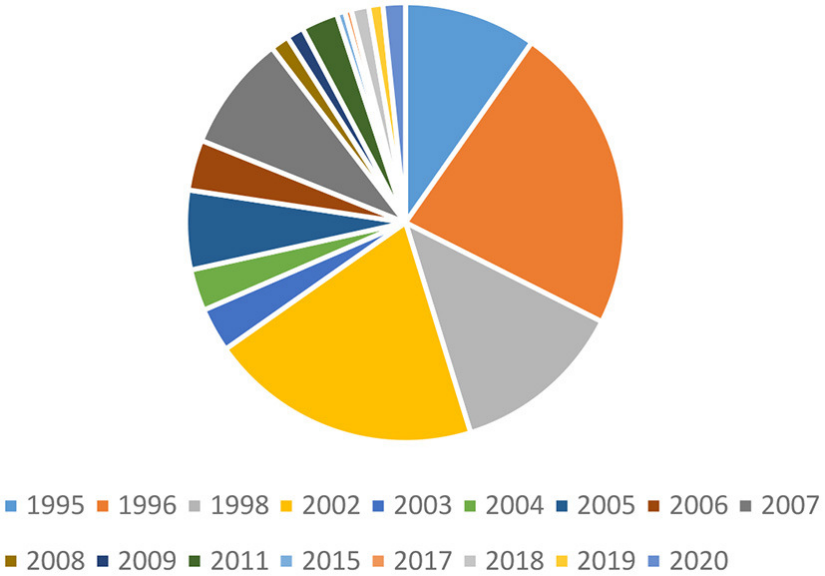
Pie chart for 1990–2022 proportion of high-quality publications.

We then further figure out the proportion of high-quality publications in total publications for each year of 1990–2021 (data source: Google Scholar) so as to learn about the quality of cultural tourism publications. Note that we define high-quality publications as those published on journals with three-star or above based on the 2021 ABS academic journal guide. According to Figure 2 (i.e., the proportion of high-quality publications for each year of 1990–2021) and Figure 3 (i.e., the corresponding pie chart of Figure 2), we can learn that despite the obvious fluctuations, the general trend for each year's proportion of high-quality publications is still going up, demonstrating that the quality of cultural tourism research is also growing continuously.

Cultural tourism is also widely discussed in various research fields. According to Table 1, cultural tourism is most frequently discussed in Economics (20.91%), followed by Management (20.29%; data source: Google Scholar). In terms of specific themes, such themes as connotations (i.e., a form of cultural consumption; e.g., Barbieri and Mahoney, 2009; Falk, 2011; Du Cros and McKercher, 2020), motivations and typology (e.g., Lee and Hsu, 2013; Chang et al., 2014; Packer and Ballantyne, 2016), cultural heritage tourism (e.g., Yankholmes and McKercher, 2015; Patuelli et al., 2016), gastronomy tourism (e.g., Everett and Aitchison, 2008; Montanari, 2009), shopping tourism (e.g., Rabbiosi, 2011; Saayman and Saayman, 2012; Choi et al., 2016b), destination branding (e.g., Josiassen et al., 2013; Barnes et al., 2014; Mariani and Giorgio, 2017), and tourism experience (e.g., Chatterley et al., 2013) are frequently discussed in extant literature. Additionally, some research also shows some interest in themes such as economic effects (e.g., Cisneros-Martínez and Fernández-Morales, 2015; Ponferrada, 2015; Noonan and Rizzo, 2017; Artal-Tur et al., 2018; Guccio et al., 2018), creative economy (e.g., Ponzini et al., 2016; Fahmi et al., 2017; Mostafanezhad and Promburom, 2018), and anthropology (e.g., Ochoa Zuluaga, 2015; Pabel et al., 2017).

**Table 1:** Proportion in various research fields.

**Research fields**	**Proportion**
Economics	20.91%
Management	20.29%
Jurisprudence	13.87%
Social Sciences	11.39%
Science	7.87%
History and Geography	5.59%
Other	4.35%
Engineering	3.73%
Technology	2.69%
Medicine	2.48%
Philosophy	2.07%
Arts and Recreation	1.24%
Literature	0.83%
Education Science	0.83%
Philosophy	0.41%
Literature	0.41%
Computer Science	0.41%
Religion	0.21%
Language	0.21%
Agricultural	0.21%
**Total**	100.00%

Generally, cultural tourism is a flourishing topic in the tourism research, which is another evidence for cultural tourism to be a trending type of tourism. In light of this, tourism organizations have to adjust their strategy and positioning to better embrace *culturedness*, thus hedging the potential risks caused by the failure to comply with the promising trend in the tourism domain.

### Definition

Thanks to the changing nature of cultural tourism, there exists no perfect definition that gives an absolutely thorough interpretation. Nevertheless, some definitions are of relatively widespread coverage and are illuminating for further identification of the notion. For example, Richards (2003, 2007) believed that cultural tourism is the movement of people toward cultural attractions, somewhere other than their habitual place of residence, to obtain information and knowledge to fulfill their cultural demands. In 2017, the UNWTO updated the definition as “A type of tourism activity where the essential motivation of tourists is to learn, discover, experience and consume both tangible and intangible cultural resources in a tourism destination” (UNWTO, 2017, p. 30). Compared to Richards's definition, the new one expands the boundary of traditional physical-heritages-based cultural resources by including intangible ones, innovatively relating cultural tourism to ways of life, creativity, and everyday culture (Richards, 2018; UNWTO, 2018). In 2018, Richards (2018) further defined cultural tourism as a collection of cultural practices engaged in by a wide range of actors, especially by tourists themselves. In light of this, it seems that cultural tourism is no longer regarded as a merely specific form of tourism or a coherent tourism market, but is attached with a new identification embracing expanding broadness and complexity (Jovicic, 2016; Richards, 2018). With regard to this issue, tourism organizations are also expected to equip with transformative sense and dynamic thinking to adapt to the changing nature of cultural tourism.

### Typology of cultural tourists

The typology of cultural tourists is also a popular topic in cultural tourism research. According to Barbieri and Mahoney (2009), cultural tourists could be either general or specific based on their degree of mixing or omnivorousness in cultural tourism behavior. Apart from a general grouping like this, cultural tourists can also be classified into specific groups or segments based on cultural experience appetites (e.g., art museum lovers, movie fans, etc.; Baltaci and Cakici, 2022), age, physical contexts (e.g., holiday type and attraction setting; Richards and van der Ark, 2013), and motivations (i.e., the most common and effective criterion; Du Cros and McKercher, 2020). For example, younger visitors tend to consume contemporary art, creativity, and modern architecture, whereas older visitors tend to be frequenters of traditional monuments and museums (Richards and van der Ark, 2013). Pearce (1982) divided the needs of tourists into five levels from low to high based on Maslow's hierarchy of needs. The higher the level of needs, the greater the sense of satisfaction felt by tourists. Correia et al. (2013) roughly divided the motivations of cultural tourists into two types: one is culture-seeking motivation, and the other is non-cultural motivation. Specifically, the former type usually includes culture learning, which is the stem of culture-seeking motivation and is frequently highlighted by scholars, while the latter, however, includes anything other than culture learning (Packer and Ballantyne, 2016).

Based on the degree of *culturedness* (Jovicic, 2016) presented by the two motivation types (i.e., cultural and non-cultural), cultural tourists can be further divided into two types: tourists who consider culture consumption as their main motivation, and those for whom culture consumption is only an alternative that is complementary, secondary, or even accidental (Galí-Espelt, 2012). Note that the degree of *culturedness* could be measured in terms of the duration of visit and the frequency of cultural experiences (Galí-Espelt, 2012; Jovicic, 2016), and that the majority of cultural tourists tend to seek a combination of learning and hedonic-entertainment consumption-related dimensions, or edutainment, a unique blend of education and entertainment, form the process of cultural tourism (Geissler et al., 2006; Kay, 2006; Ballantyne and Packer, 2016; Jovicic, 2016).

Given that the diversity and complexity of cultural tourists are constantly increasing, tourism organizations are responsible to set up diverse functional departments to cover various demands of tourists up to the hilt.

### Important sub-themes and branches

This section discusses several flourishing sub-themes or branches in cultural tourism research. In addition to cultural heritage tourism, which is the most lineal branch of cultural tourism research, this section also includes gastronomy tourism and shopping tourism, two promising cultural tourism sub-themes developed by the prosperous culturalization process in gastronomy and shopping domain (Timothy, 2011; Báez-Montenegro and Devesa-Fernández, 2017; Redondo-Carretero et al., 2017; Richards, 2018).

#### Cultural heritage tourism

Originating from tourism managers' business instinct that cultural heritage can be a driver of tourism development, cultural heritage tourism is regarded as the most lineal branch of cultural tourism (Smith, 2015; Gravari-Barbas, 2018). As Timothy (2011) put it, cultural tourism and heritage tourism have minimal distinction. According to Park (2013), cultural heritage tourism is defined as visits or experiences of physical or immaterial relics of the past. It is also noted as a product of cultural patrimony that contains even smallest piece of existing cultures such as folkways and everyday scenes (Timothy and Nyaupane, 2009; Richards, 2018).

Extant literature on cultural heritage tourism tend to show great interest in list of World Heritage Sites (WHS), especially in terms of the negative side. For example, Frey and Steiner (2011) are skeptical to the rationality of WHS list. They argued that the benefits of a WHS designation is limited to cultural destinations that fail to generate fair notability and related developmental resources, rather than to destinations that are already well-known and flourishing. Alberts and Hazen (2010) also questioned the credibility of the criteria for WHS designation by pointing out that these criteria could lead to ambiguous definitions and varying interpretations in different cultural settings. In addition, Patuelli et al. (2016) suggested that the existence of WHS list can be a primary fuse for an even fiercer inter-destination competition.

The conservation, preservation, and protection of cultural heritage is also a popular topic in cultural heritage tourism research. For example, Hall et al. (2016) identified that cultural landscapes, local rituals, built environments, buried archeology, and climate changes could be potential threats for the conservation of cultural heritages. They also proposed that strategies like planning time-scales, monitoring, management, maintenance, loss and obsolescence could be effective to deal with these challenges. By analyzing real-life cases of Hong Kong and Singapore, Li (2003) focused on the contradictions between the conservation and change of cultural heritage and concluded that the balance point between the two depends to the actual impacts (i.e., cultural or economic returns vs. residents' life quality) brought by the cultural heritages.

Other than the aforementioned two issues (i.e., WHS list and conservation), many researchers also pay attention to other issues in the development of cultural tourism. For example, Gravari-Barbas (2018, p. 3) concerned that tourism might become a “heritage-production machine,” as heritage tourism is tourist-centered and is subject to tourists demands (Urry, 1992; Yang, 2011; Park, 2013; Dela Santa and Tiatco, 2019). Zhang (2022) argued that more and more local residents suffered from the over-tourism in their homelands and that the elimination of local perspectives and livelihoods may result in emigration and depopulation, which eventually affects the cultural site's viability. Generally, people are increasingly concentrating on the trade-off between further development and long-term sustainability.

According to this section, cultural heritage tourism, which is the stem of cultural tourism, is usually associated with various professional staff such as museum commentators, historic buildings maintenance engineers, and archaeologists etc. In light of this, tourism organizations are obliged to optimize personnel management through professional training, precise selection, and scientific evaluation.

#### Gastronomy tourism

Gastronomy tourism is known to be one of the most important sub-themes of cultural tourism research, for it is believed to be of much *culturedness* (Jovicic, 2016). For most tourists, food is not only sustenance, but also a cultural artifact with a myriad of facets that can be enjoyed in various locations and through many different activities (e.g., food trails, events, and festivals; Everett and Aitchison, 2008). As Cohen (1992) put it, tourist cuisines are by nature new and sui generis cultural products whose tourists-adaptive transformation is not only mere fusion or hybridization of exotic and local elements but also a multidirectional and multidimensional innovation or creation. Meneguel et al. (2019) also suggested that gastronomy tourism is usually acknowledged as a cultural practice where typical cuisines are treated as sensory and experiential heritage and that the emergence of gastronomy tourism revitalizes and diversifies tourism, thus promoting gastronomy culture, multi-ethnicity, and global exposure. Another evidence for the cultural influence of gastronomy tourism is that it is by nature the swelling of cultural food consumption (Montanari, 2009). As heritage unites with ritualistic elements, cultural food consumption, a symbolic portrayal of its root culture, helps to arouse the connection between individuals and their own cultural heritage (Horng and Tsai, 2010; Wu et al., 2021). Additionally, such factors as dining atmospherics (Ha and Jang, 2010; Jang et al., 2012; Jang and Ha, 2015), employees' appearance or ethnicity (Baker and Kim, 2018), the presence of other customers (Song et al., 2019), menu design (Kim et al., 2017; Yu et al., 2020), and service innovation (Su, 2011) will potentially influence consumers' cultural food consumption experiences, thus increasing the *culturedness* of gastronomy tourism (Sims, 2010).

Throughout the 30-year research history of gastronomy in the tourism domain (Okumus, 2021), gastronomy tourism has been acknowledged as a unifier in terms of multiple perspectives. In terms of major contents, gastronomy tourism, interchangeably named culinary tourism, food tourism, or taste tourism in some studies (Okumus et al., 2018), is the combination of understanding, savoring, and consuming the local food and culture in tourist destinations and is usually associated with cuisine, gourmet, tasting, and wine tourism (Smith and Xiao, 2008). Similarly, Henderson (2009) defined gastronomy tourism as the binding agent of a series of intentional and exploratory participating behaviors in gastronomy activities, such as food preparation, food display, cooking, and dining. In terms of involved parties, gastronomy tourism is also known as the bond between the primary and secondary food producers, food festivals, restaurants, and local food tasting sites (Hall and Mitchell, 2000). In terms of its relevant domains, gastronomy tourism “represents a multifaceted research area rising in prominence” with the potential to function as an instrument that regenerates “academic research to the forefront of geographical theory, tourism policy, cultural studies, and sociological analysis” (Everett and Aitchison, 2008, p. 151).

In more recent research on gastronomy tourism, scholars tend to pay attention to such topics as gastronomy tourism experience, scale and measurement, and luxury gastronomy tourism. For example, many related studies tend to explore gastronomy experience in tourism through cognitive evaluations (Berbel-Pineda et al., 2019; Sthapit et al., 2019; Lai et al., 2021), although Richards (2021) argued that the literature on gastronomy experience has shifted from a cognitive to an emotive approach. Specifically, a small group of scholars (Williams et al., 2019; Mohamed et al., 2020; Promnil, 2021) got to realize that affective gastronomy experience turns out to be a stronger inspirer for tourists' satisfaction and behavioral intention than cognitive attributes do. In terms of scale and measurement, Bastiaansen et al. (2019) proposed a scale to measure local food preference, inspired by which Hsu et al. (2022) created a valid and reliable scale for measuring tourists' affective gastronomy experiences (TAGES) in tourism destinations. As for luxury gastronomy tourism, Balderas-Cejudo et al. (2022) found it a top type of gastronomy tourism and that its impacts on local development are usually shown through local Michelin-starred restaurants.

Thanks to the dominant role played by taste (i.e., one of the important involved senses) in tourists' ecological body-environment exchange, gastronomy, a burgeoning subfield of sociological and anthropological research (Beardsworth et al., 2002), became a crucial precondition (Hall and Sharples, 2008), a leading attraction (Dann, 1996), and one of the most important components of tourism activities (MacCannell, 1973; Urry, 1990; Cohen and Avieli, 2004). Meanwhile, as an indispensable branch of cultural tourism, gastronomy tourism proves to be economically (Okumus et al., 2007), environmentally (Hjalager and Richards, 2002), sociologically (Richards, 2002), and psychologically (Graburn, 1977; Cohen, 1986; Quan and Wang, 2004; Everett, 2008; Henderson, 2009; Björk and Kauppinen-Räisänen, 2017; Ellis and Mattison Thompson, 2018) influential. In light of this, gastronomy tourism is expected to keep on flourishing (Hsu et al., 2022) and continue to be a priority of tourism and hospitality research in the next 75 years (2020–2095) (Okumus, 2021) despite the worldwide health crisis. As [(Everett, 2019), p. 9] put it, “Food tourism research is still very much on a journey and has much still to offer, therefore I urge scholars to consider adding new empirical contributions which analyze new aspects of this form of tourism activity.”

#### Shopping tourism

Shopping tourism is another flourishing sub-theme of cultural tourism, for shopping is a common cultural activity among those forces driving the choice of tourism destinations, especially in cultural tourism destinations where cultural creative economy is prosperous and booming (Saayman and Saayman, 2012). As a direct sociocultural experience, shopping is a mediator through which tourists manage to develop familiarity with the unique culture and distinct features of the target tourism destinations, thus improving tourists' satisfaction and pleasure (Rabbiosi, 2011; Way and Robertson, 2013; Choi et al., 2016a; Sun et al., 2017). When visitors are in shopping places, they can get in close touch with the cultural connotations of tourist destinations as well as create unique travel experiences of their own through interactive behaviors such as information search and counter-offer behavior (Liu et al., 2019).

Initially proposed by Jansen-Verbeke (1991), shopping tourism got its first definition as a tour with the main purpose of purchasing products from Timothy and Butler (1995). It is then further defined as a specialized tour in which tourists tend to spend more than 50% of net tourism expenses (i.e., total tourism expenses excluding lodging and transportation costs) on pure shopping activities (Michalkó and Varadi, 2004; Timothy, 2005; Saayman and Saayman, 2012; Jin et al., 2020). In more recent years, shopping tourism, combining both tangible (i.e., tangible consumer goods such as souvenirs and gifts) and intangible (i.e., intangible touristic experiences) stimulations, is defined as a contemporary form of tourism fostered by individuals for whom purchasing goods outside of their usual environment is a determining factor in their decision to travel (UNWTO, 2015; Sharma et al., 2018; Jiang and Yu, 2020).

In addition to its *culturedness* and definition, extant literature on shopping tourism is also frequently focused on such topics as attributes of shopping tourism (Baker et al., 1988, 2002; Henderson et al., 2011; Albayrak et al., 2016; Lee and Choi, 2020), cross-border shopping (Yeung et al., 2004; Wong and Wan, 2013), souvenir shopping (Masset and Decrop, 2021), passengers' shopping motivations (Lin and Chen, 2013), duty-free shopping (Han and Hyun, 2018), the typology of shopping tourists (Choi et al., 2016a), and shopping risk management (Hsieh and Tsao, 2014). In addition, Kattiyapornpong and Miller (2012) found that shopping tourism could function as one of the competitive advantages of tourism destinations, thus bringing tremendous economic benefits to local development. Lee and Choi (2020) examined the effects of asymmetric shopping tourism attributes on shopping destination satisfaction levels and designed two pyramid-shaped figures prioritizing both shopping-specific attributes and destination-specific attributes based on the categorization of their asymmetric impacts. Choi et al. (2018) examined the trust in the shopping destination as an antecedent of shopping tourists' perceptions of value based on an RFT framework. Correia et al. (2019) dug deep into Chinese tourist perceptions of luxury shopping visits to Hong Kong to explore the nature of luxury buying behavior.

According to Section Gastronomy tourism and Section Shopping tourism, gastronomy and shopping, the two representative components of general tourism, are both on their way to become part of cultural tourism through the process of culturalization. Therefore, it is necessary for tourism organizations to take on the culture-oriented strategy so as to conform to the inevitable trend of culturalization.

### Optimization of the cultural tourism development

This section reviews literature on the optimization of cultural tourism development, including destination branding and consumer experience, two significant tools to optimize cultural tourism development.

#### Destination branding in tourism

Branding of tourism destinations is an essential tool for tourism destinations to attract more tourists and thus to flourish. By far, there is barely any research specifically focusing on destination branding in cultural tourism. However, destination branding in general tourism has generated a bunch of attention from scholars, from which we can capture some of the developmental patterns of destination branding in cultural tourism.

As the market competition intensifies, more and more companies and organizations get to realize that tourist destinations also need to make the most of such valuable assets as their developed brands to position and differentiate themselves properly (Aaker, 2009). Therefore, tourism branding, usually known as destination branding, has become a key theme for tourism researchers since the late 1990's (Liu et al., 2020). For example, Konecnik and Gartner (2007) explored the effectiveness of customer-based destination brand equity (CBDBE) and proposed a comprehensive evaluation system consisting of four dimensions: image, awareness, quality, and loyalty. Furthermore, the determinants of CBDBE have been identified from three main perspectives: tourists' travel-related factors (e.g., destination experience and consumption social visibility); DMOs' branding-related factors (e.g., DMOs' cooperation or power); and resident-related factors (e.g., tourist–resident interaction; Marzano and Scott, 2009; Josiassen et al., 2013; Barnes et al., 2014; Mariani and Giorgio, 2017). Applying traditional research methods of brand image and brand personality to tourism destinations, Hosany et al. (2006) found that destination image is closely related to destination personality and that the emotional component of destination image captures the majority of variance on destination personality dimensions. Taking the Gold Coast in Australia as a case study, Marzano and Scott (2009) concluded that a lack of unity and collaboration amongst stakeholders could barely affect the positive outcomes brought by a destination branding process. The authors also provided a detailed inventory of the interests advanced by stakeholders' power in the forms of persuasion and authority in a branding process.

In the past decade, the connotation of destination branding is becoming increasingly comprehensive. According to Campelo et al. (2014), destination branding is meant to combine a tourism destination's environmental, social, and cultural capital, thus creating a unique and differential image of the destination. Pike and Page (2014) proposed that DMOs should also include local residents (other than visitors) into their target audience base during destination branding. Additionally, destination brand image is no longer an exclusive creation of DMOs alone, but a complex co-creation of thousands of social media users who tend to share their traveling experiences and feelings online (Lo et al., 2011; Mak, 2017). In light of this, scholars began to explore the potential use of mainstream social media platforms (e.g., Instagram) for destination branding purposes (Fournier and Avery, 2011; Fatanti and Suyadnya, 2015; Oliveira and Panyik, 2015). When it comes to the connection between consumers and destination branding, scholars tend to focus on destination brand love (DBL), which is usually defined as a multi-dimensional construct including passionate love, emotional attachment, self-brand integration, and self-brand identification in the context of tourism (Tsai, 2014; Lee and Hyun, 2016; Aro et al., 2018; Zhang et al., 2020). Through manifesting their love toward certain destination brands, tourists manage to express their consumer identity and personality, getting closer to their ideal self (Batra et al., 2012; Belk, 2014).

From the year 2020 on, research on destination branding has become more detail-oriented and innovative when a great many scholars tend to focus on topics that are either further extensions of existing ones or accumulative innovations. For example, further to the series of research on mainstream social media platforms for destination branding purposes, Filieri et al. (2021) explored how destination brand love (DBL) is expressed on Instagram using a mixed-methods approach. Pan et al. (2021) innovatively explore destination brand image from the perspective of gender, contributing greatly to the measurement scale development of destination gender, which is an important topic in destination branding literature. Considering New Zealand as a space tourism destination, Scott (2022) proposed that the destination branding of New Zealand should be updated with the development of the society to include cultural factors, geopolitical factors, and sustainability-related factors.

#### Consumer experience in cultural tourism

Consumer experience, a central concept in marketing (Pine et al., 1999), is also a pivotal construct in the tourism industry and is usually known as tourism experience under touristic context (Otto and Ritchie, 1996; Sørensen and Jensen, 2015). Extant literature on tourism experience has proved that the key to retain flow of tourists is to create a memorable tourism experience for them, which is also applicable for cultural tourism.

Many researchers have long been working on exploring the essence of tourism experiences. According to Chen et al. (2014), a tourism experience is different from general consumer experiences in that it is strongly driven by service and hospitality. It usually occurs in distinct stages including at-home stage, at-the-destination stage, and after-returning stage (Chatterley et al., 2013). In as early as 1979, Cohen found that the ultimate tourism experience could be compared as a religious experience or the result of pilgrimage, where tourists search for something less tangible than the trip and more rewarding than just being there (Vallee, 1987). Mannell and Iso-Ahola suggested in 1987 that the core of leisure tourism experience is flow experience and that the search for the ultimate tourism experience is a quest for authenticity, center, meaning, or values. From the perspective of psychology, the main benefits of leisure tourism experiences emanate from the interplay of two forces: escaping (of routine and stressful environments) and seeking (for recreational opportunities of certain intrinsic rewards). However, it remains a problem to neatly distinct plain tourism experience from leisure experience (Mannell and Iso-Ahola, 1987).

As the famous tourism anthropologist Cohen (1979) put it, different tourists need different tourism experiences, which is of special significance for tourists. For example, excitement-seeking tourists tend to show a special preference for adventure tourism, while food lovers turn out to be loyal customers of gastronomy tourism (Gyimóthy and Mykletun, 2004; Konu, 2015; Sthapit, 2019). However, due to factors such as heterogeneity in customer preferences, uncertainties in destination choices, and contingencies related to group activities (Hsiao et al., 2015), tourism experience proves to be a quite complex concept that is neither definition-friendly nor manipulation-friendly (Mei, 2014). In light of this, many tourism firms begin to introduce co-creation, a tourism designing mode in which tourists function as independent co-creators rather than passive consumers to create value in interactive consumer-firm relationships (Dalonso et al., 2014; Prebensen et al., 2017; Geng et al., 2022; Sun et al., 2022). In a high co-creation context, tourist wellbeing increases with increased active participation (Björk, 2014). Moreover, thanks to all the individual resources devoted to the co-creation of tourism experience, psychological ownership also comes into play and enhances tourists' subjective value, thus stimulating tourists' loyalty (i.e., intentions to re-visit; Prebensen et al., 2013; Sugathan and Ranjan, 2019).

Tourism experience is usually recognized as a psychological phenomenon where expectations, individual sensing and perception, and memorizing are crucial components (Larsen, 2007). Theoretically, the boundary of tourism experience can be extended to various psychological fields such as cognitive psychology and social psychology. For example, research on experiential consumption believes that from the perspective of tourist psychology, it is the experience of shopping at a tourism destination, rather than the physical goods purchased, that really matters in tourism consumption. Furthermore, as MacCannell (2002) put it, a tourism experience is by nature an experiential commodity whose value is legitimated by consumers' pursuit of ego fulfillment. Therefore, the essence of tourism consumption is also experiential consumption (Moldes et al., 2019; Gilovich and Gallo, 2020; Puente-Díaz and Cavazos-Arroyo, 2021). Experiential consumption is a concept originally proposed by Van Boven and Gilovich (2003). Using the experience sampling method, Kumar et al. (2020) found that experiential shopping would lead to more instant happiness, which is a sense of happiness perceived by consumers from a certain consumption behavior (Dunn and Weidman, 2015). Experiential consumption will also lead to more positive emotions (Carter and Gilovich, 2010; Barton et al., 2019), more jealousness (Lin et al., 2018), more interactive behavior (Chen et al., 2021), and fewer regrets (Rosenzweig and Gilovich, 2012).

According to Section Destination branding in tourism and Section Consumer experience in cultural tourism, extant literature on cultural tourism development has been increasingly emphasizing the power of people. Specifically, optimizing destination branding and promoting tourist experience are both effective methods centered on destination-tourist bonding. In light of this, tourism organizational change in the future may as well center on people and thus pass on to the promising humanity-centered times.

### Summary of interconnections

Combining all the sub-interconnections mentioned in the last paragraph of each section, the general interconnections between the review of cultural tourism research and the change of tourism organizations can be summarized as: tourism organizations are interconnected with cultural tourism research in terms of changes in strategy, structure, personnel management, and organizational culture, and thus are obliged to implement multi-facet changes to adapt to the volatile tourism industry. Table 2 summarizes and reviews all the aforementioned interconnections for quick check.

**Table 2:** Summary of interconnections.

**Sections**	**Conclusions of each section**	**Interconnections with tourism organizational change**	**Aspects of interconnections (in terms of…)**
Current status of cultural tourism research	Cultural tourism is a flourishing and trending type of tourism.	Tourism organizations have to adjust their strategies to fit in the changing context.	Organizational strategy
Definition	The connotation of cultural tourism is increasingly broad, complex, and volatile.	Tourism organizations must equip with transformative sense and dynamic thinking to adapt to the changing nature of cultural tourism.	Organizational strategy
Typology of cultural tourists	The diversity and complexity of cultural tourists are increasing.	Tourism organizations are responsible to set up diverse functional departments to cover various demands of tourists up to the hilt.	Organizational structure
Cultural heritage tourism	As the stem of cultural tourism, cultural heritage tourism is usually associated with various professional staff.	Tourism organizations are obliged to optimize personnel management through professional training, precise selection, and scientific evaluation.	Personnel management
Gastronomy tourism and Shopping tourism	The two representative components (i.e., gastronomy and shopping) of general tourism are both on their way to culturalization.	Tourism organizations have to take on the culture-oriented strategy so as to conform to the inevitable trend of culturalization.	Organizational strategy
Optimization of the cultural tourism development	Extant literature on cultural tourism development has been increasingly emphasizing the power of people.	Tourism organizational change in the future may as well center on people and thus pass on to the promising humanity-centered times.	Organizational culture
In summary	Tourism organizations are interconnected with cultural tourism research in terms of changes in strategy, structure, personnel management, and organizational culture, and thus are obliged to implement multi-facet changes to adapt to the volatile tourism industry.		

## Implications for tourism organizational change

Based on the systematic literature review of cultural tourism research presented in Part 2 as well as its interconnections with tourism organizational change (as is shown in Table 2), several irradiative implications could be proposed for tourism organizations to implement feasible changes. In this part, all the potential implications will be presented section by section and will be further summarized into Table 3.

**Table 3:** Implications for tourism organizational changes.

**Types of changes**		**Rationales for changes**	**Details of changes**	
		Cultural tourism becomes a mainstream tourism type in the global tourism market.		
Culture-based strategic change		Most tourists travel for cultural motivations.	Tourism Organizations should adopt a culture-based strategy (i.e., to further develop cultural tourism products) instead of the traditional sightseeing-based strategy.	
		Traditional tourism factors such as gastronomy and shopping have also been attached with some *culturedness*.		
Personnel-based structural change		The connotation of cultural tourism is increasingly broad and complex.	Tourism organizations should launch a structural change through personnel change techniques such as proper new hiring, professional training, and rearrangement of responsibility and role.	
		Cultural tourists have complex and diverse motivations for traveling.		
		Cultural tourism research has been widely discussed in various academic disciplines.		
		Tourism organizations have to increase the diversity and subdivisions of their departments to adapt to the ever-complex cultural tourism connotation.		
	Tourist-oriented communication change	Tourists are the final examiners and evaluators of tourism organizational change performances.		
		Tourist-oriented destination branding techniques are more likely to attract a steady flow of tourists. The quality of tourist experience decides the development of tourism destinations.	Tourism organizations should launch a tourist-oriented communication change through adopting fascinating destination branding techniques, co-creating memorable tourism experiences, and seeking for proper balance between authenticity and glocalization.	Tourism organizations are obliged to respect and take advantage of the value brought by people, including tourists and employees, thus prioritizing humanity within their organizational culture.
		The authenticity of endemic culture also needs to appeal to appetites of tourists.		
Humanity-centric organizational culture change		Tourism organizations should create and strengthen an omnidirectional connection with tourists to better capture their changeable demands and preferences.		
	Employee-centric emotion management	Employees' emotions toward change usually represent organizational change readiness and capacity.	Tourism organizations should optimize their employee-centric emotion management and thus to equip themselves with sustainable resilience, secure readiness, and excellent capabilities to successfully implement daily changes.	
		Employee-centric emotion management determines change success rate.		

### Culture-based strategic change

Above all, it is inevitable for tourism organizations to change their traditional sightseeing-based strategies into culture-based ones. For one thing, cultural tourism, instead of traditional tourism, is gradually becoming a mainstream tourism type in the global tourism market (UNWTO, 2018, 2021). According to the aforementioned Figures 1, 2, cultural tourism research is becoming increasingly flourishing, which turns out to be another evidence for the growing status of cultural tourism. For another thing, traveling motivations of most tourists are more or less of some *culturedness* (Jovicic, 2016) when traditional non-cultural motivations such as relaxation-seeking, sports-seeking, family-relationship-seeking, pressure-escaping, autonomy (freedom)-seeking (Özel and Kozak, 2012), identity (achievement)-seeking (Bond and Falk, 2013), and novelty-seeking (Sugathan and Ranjan, 2019; Prayag et al., 2021; Pereira et al., 2022) are increasingly being reduced to appendants of cultural motivations. For example, gastronomy, which has long been acknowledged as a traditional mainstream touristic motivation way off cultural stuff, is now universally accepted as a cultural motivation by tourists (Cohen, 1992; Everett and Aitchison, 2008; Jovicic, 2016). In addition, the swelling of cultural food consumption is gradually driving gastronomy tourism to become an important sub-sector of cultural tourism as well as an inevitable component of general tourism (i.e., gastronomy is a necessary component of all types of tourism activities; Montanari, 2009; Sims, 2010). Similar mechanism also applies in shopping, the closest companion of tourism (Jansen-Verbeke, 1991). The boom of cultural souvenir shopping and tourists' evergreen shopping enthusiasm toward cultural intellectual properties (IP) are robust evidence for the prevailing sense of *culturedness* in touristic shopping activities (Masset and Decrop, 2021). Therefore, tourism organizations, by launching a strategic change where culture-based strategy substitute for traditional sightseeing-based strategy, should quickly adapt to the cultural revolution in the tourism industry to survive the volatile future.

### Personnel-based structural change

Thanks to the increasing complexity of cultural tourism connotation, tourism organizations are also subject to adaptive structural change that is focused on personnel management. According to Richards (2018), the connotation of cultural tourism is increasingly focused on the pursuit of broadness by including concepts such as in-betweenness and non-tourism. The expanding broadness of cultural tourism connotation can also be reflected by diverse typologies of cultural tourists. Specifically, cultural tourists have been classified upon factors such as personal appetite (Barbieri and Mahoney, 2009; Baltaci and Cakici, 2022), age (Richards and van der Ark, 2013), physical contexts (Richards and van der Ark, 2013; Richards, 2018), and motivations (Pearce, 1982; Correia et al., 2013; Jovicic, 2016; Packer and Ballantyne, 2016; Du Cros and McKercher, 2020). As Russo and Richards (2016) put it, cultural tourists can no longer be regarded as static categories when most actors engaged in the process of cultural tourism begin to perform different roles relative to one another. Additionally, the increasing broadness and complexity of the connotation and typology enhance the connection between cultural tourism and some major academic disciplines such as management, sociology, economics, anthropology, and psychology, which is consistent with our finding that cultural tourism has been widely discussed in various research fields (see Table 1). In light of this, tourism organizations are obliged to launch a structural change by increasing the diversity and subdivision of their departments, thus adapting to the increasing complexity of the newly adopted culture-based strategy.

More importantly, structural changes are usually accompanied with personnel management such as new hiring, professional training, rotation of role and responsibilities, and scientific performance evaluation (Graetz and Smith, 2010). By putting together a group of selected employees with enough expertise, position power, credibility, and leadership skills, proper personnel changes potentially solidify structural changes that took place in tourism organizations (Kotter, 1995; Thomas et al., 2011). For example, hiring professional chefs to guarantee the authenticity of local ethnic restaurants (Cohen and Avieli, 2004; Chatzopoulou et al., 2019; Meneguel et al., 2019; Yu et al., 2020), training restaurant employees to improve service awareness by removing the order barrier of tourists (Lai et al., 2019; Yu et al., 2020), and properly arranging and balancing responsibilities of different departments to gather joint efforts (Smith and Xiao, 2008), are all feasible ways for tourism organizations to make structural changes in preparation for the adoption of gastronomic culture-based strategies. In general, tourism organizations are suggested to make personnel-based structural changes to guarantee their readiness and capacity and thus to further consolidate their newly adopted culture-based strategy.

### Humanity-centric organizational culture change

According to Section Destination branding in tourism and Section Consumer experience in cultural tourism, methods that are adopted to optimize the development of cultural tourism are increasingly centered on the importance of people. For example, more and more consumer psychological techniques are applied to destination branding when improving consumer experience becomes the key to attract a steady flow of tourists in most destinations. As Feldman (2000) put it, human agents are the initial source and trigger for all the potential development and changes. Therefore, tourism organizations are obliged to respect and take advantage of the value brought by people, including tourists and employees, thus prioritizing humanity within their organizational culture.

#### Tourist-oriented communication mechanism

Tourism organizations are first obligated to develop a tourist-oriented communication mechanism, for the final performances of organizational change are inclined to be examined and evaluated by consumers (i.e., tourists). A successful tourist-oriented communication mechanism is usually associated with fascinating destination branding (Aaker, 2009), memorable tourism experience (Sørensen and Jensen, 2015), and proper balance between authenticity and glocalization (Soulard et al., 2019). Firstly, tourism organizations should adopt more tourist-oriented techniques such as arousing destination brand love (Lee and Hyun, 2016), inspiring self-brand integration (Tsai, 2014), as well as taking advantage of tourists' respect and trust toward authoritative titles (e.g., the list of World Heritage Sites; Jimura, 2011) to carry forward the brand of a target destination, thus strengthening the tourists-destination connection (Aro et al., 2018; Zhang et al., 2020). Secondly, tourism organizations should also create a memorable tourism experience, which proves to be the key to attract a steady flow of tourists (Mei, 2014), through bonding techniques such as encouraging co-creation of tourists (Dalonso et al., 2014; Prebensen et al., 2017; Geng et al., 2022; Sun et al., 2022). Thirdly, endemic tourism destinations should maintain the balance between authenticity and glocalization to deal with the dilemma caused by tourists' curiosity toward a different culture and their limited tolerance toward a strange taste. For example, some endemic restaurants in tourism destinations try to slightly adapt their traditional cuisines to the taste of ecdemic tourists, thus bringing the authentic culture of the tourism destination and simultaneously improving tourists' dining satisfaction (Cohen and Avieli, 2004; Soulard et al., 2019). Therefore, tourism organizations are recommended to launch a tourist-oriented communication change to better capture tourists' changeable demands and preferences.

#### Employee-centric emotion management

Tourism organizations are also obliged to properly manage employees' emotions toward upcoming organizational changes, which prove to be essential to the success of change implementation (Errida and Lotfi, 2021). According to Al-Haddad and Kotnour (2015), the general success rate of organizational change has long been under 30%, indicating a sustained need to improve the success rate by minimizing destructive barriers such as employees' negative emotions and attitudes toward change (Rafferty et al., 2013). According to most literature on change management, employees' emotions toward change, usually known as the change motivation of employees, are inclined to represent organizational change readiness and capacity (Project Management Institute, 2013; Combe, 2014; Alwheeb and Rea, 2017). As employees usually function as the active impellers of changes (Mento et al., 2002), their change motivations and emotions, which are usually influenced by each interest-based personnel reshuffle during organizational change (Shah et al., 2016), turn out to be determinants of the change success rate (Fernandez and Rainey, 2006; Hodges, 2017). Therefore, it is vital for tourism organizations to optimize their employee-centric emotion management and thus to equip themselves with sustainable resilience, secure readiness, and excellent capabilities to successfully implement daily changes.

Additionally, these implications have been summarized into Table 3 for quick check.

## Conclusion

This study, based on a brief review addressing the tremendous expansion of cultural tourism scholarship that has evolved into a well-defined research field incorporating multi-disciplinary perspectives, manages to figure out several feasible implications for tourism organizations to implement proper organizational changes for adaptative development. According to the evolutionary process from fragmentation to systematization in cultural tourism research (Richards, 2018), cultural tourism is continuously growing from a niche market consisting of intellectuals and high-income tourists toward a mass-market open to a much wider range of people, becoming a promising trend that directs future change implementations of tourism organizations (Noonan and Rizzo, 2017). In general, this study, based on a brief review of cultural tourism research that illustrates its interconnections with organizational changes, has figured out several implications in terms of changes in organizational strategy, organizational structure, personnel management, and organizational culture. Therefore, tourism organizations are recommended to pursue *culturedness*, accompanied by corresponding means to continuously implement proper changes to hedge the potential volatility in future development.

## Data availability statement

The original contributions presented in the study are included in the article/supplementary material, further inquiries can be directed to the corresponding author.

## Author contributions

ZZ: writing and final proofreading. MG: data collection, analysis, and producing figures and reference list. All authors contributed to the article and approved the submitted version.

## Conflict of interest

The authors declare that the research was conducted in the absence of any commercial or financial relationships that could be construed as a potential conflict of interest.

## Publisher's note

All claims expressed in this article are solely those of the authors and do not necessarily represent those of their affiliated organizations, or those of the publisher, the editors and the reviewers. Any product that may be evaluated in this article, or claim that may be made by its manufacturer, is not guaranteed or endorsed by the publisher.
